# Supplementary material for: Water-Soluble Pd Nanoparticles for the Anti-Markovnikov Oxidation of Allyl Benzene in Water
Source: Nanomaterials (Basel). 2023 Jan 14;13(2):348. doi: 10.3390/nano13020348 (PMC9866704; doi:10.3390/nano13020348)
Supplement: Supplementary file 1 [file nanomaterials-13-00348-s001.zip › nanomaterials-2095306-supplementary.pdf]

Supporting Information For:

Water-Soluble Pd Nanoparticles for the Anti-Markovnikov Oxidation of Allyl Benzene in Water

Edwin Avila, Christos Nixarlidis, and Young-Seok Shon*

Department of Chemistry and Biochemistry, California State University Long Beach, 1250 Bellflower Blvd., Long Beach, CA 90840, Unites States

For correspondence: Email: ys.shon@csulb.edu. Phone: 562-985-4466.

I. ^{1}H NMR characterization of sodium ω-carboxylate-S-hexyl thiosulfate and sodium S-(5-trimethylammonio)pentyl thiosulfate ligands

II. Characterization of water-soluble Pd nanoparticles

III. Analysis of Pd nanoparticle catalytic assays

I. ^{1}H NMR characterization of sodium ω-carboxylate-S-hexyl thiosulfate and sodium S-(5-trimethylammonio)pentyl thiosulfate ligands

The ^{1}H NMR result of ω-carboxylate-S-hexyl thiosulfate sodium salt ligand is shown in Figure S1. All the chemical shifts are characteristic of the ω-carboxylate-S-hexyl thiosulfate ligand. The absence of any other peaks corresponding to the precursor, 6-bromohexanoic acid, indicates the purity of the synthesized ligand. ^{1}H NMR (400 MHz, D_2O): δ 3.13 (t, 2H, $CH_2S_2O_3^-$), δ 2.42 (t, 2H, CH_2COO^-), δ 1.81 (m, 2H, CH_2), δ 1.579 (m, 2H, CH_2), and δ 1.48 (m, 2H, CH_2).

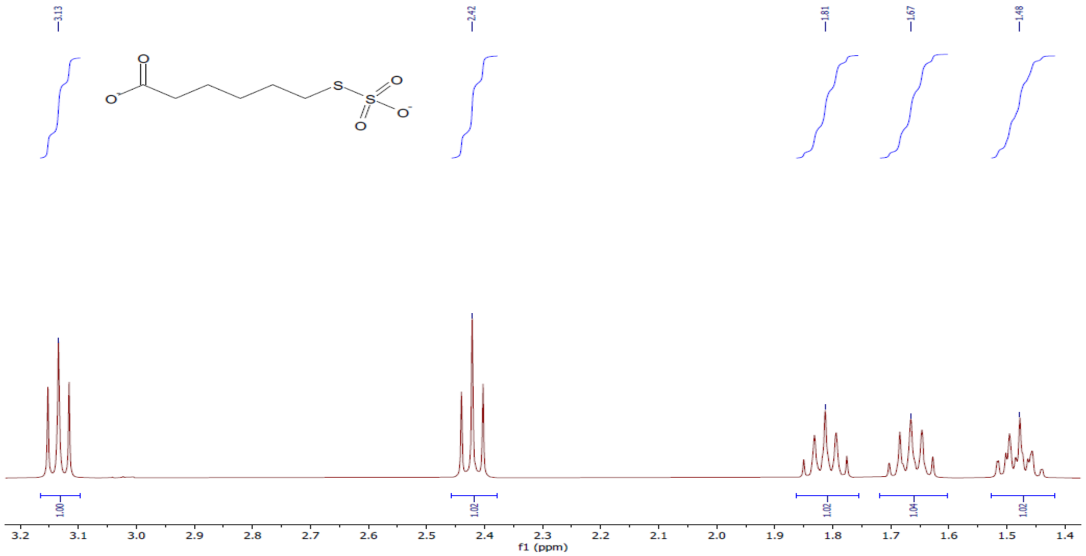

Figure S1. ^{1}H NMR spectrum of sodium ω-carboxylate-S-hexyl thiosulfate ligand.

The ^{1}H NMR of S-(5-trimethylammonio)pentyl) thiosulfate ligand is shown in Figure S2. All the chemical shifts are characteristic of the S-(5-trimethylammonio)pentyl thiosulfate ligand. The absence of any other peaks corresponding to the precursor, (5-bromopentyl) trimethylammonium bromide indicates the purity of the synthesized ligand. Note that there is a significant overlap for signals δ 3.03 - 3.08, which resulted in a complex integration (a total of

~11H). ^{1}H NMR (400 MHz, D_2O): δ 3.27 (t, 2H, $CH_2N(CH_3)_3$), δ 3.06 (t, 2H, $CH_2S_2O_3^-$), δ 3.05 (s, 9H, $N(CH_3)_3$, δ 1.79 (tt, 2H, CH_2), δ 1.79 (tt, 2H, CH_2), and δ 1.44 (tt, 2H, CH_2).

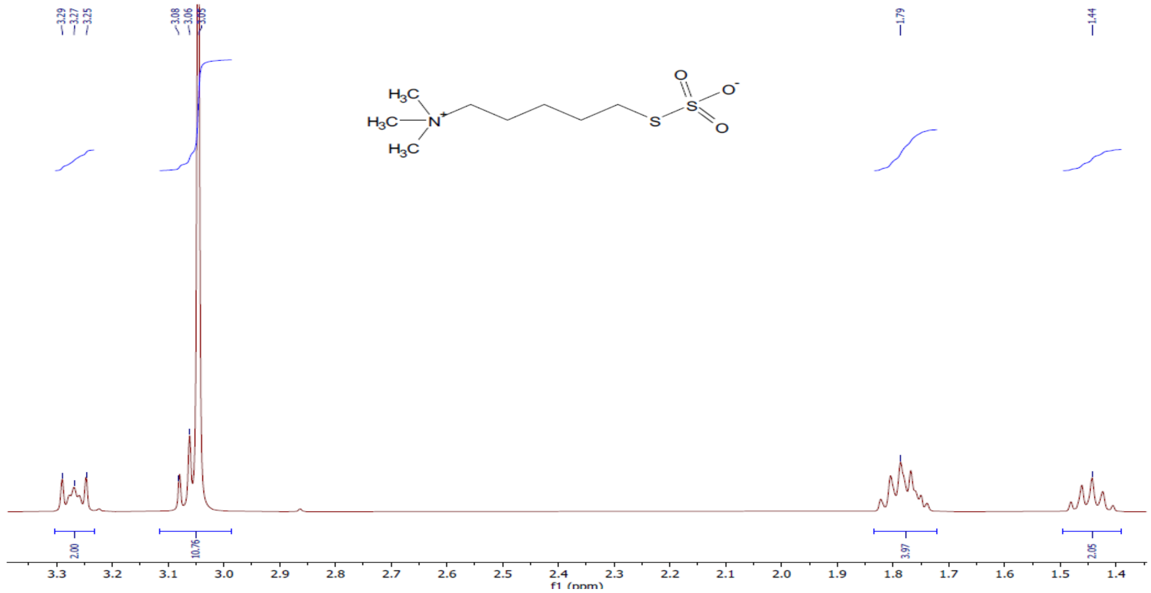

Figure S2. ^{1}H NMR spectrum of sodium S-(5-trimethylammonio)pentyl thiosulfate ligand.

II. Characterization of water-soluble Pd nanoparticles

The ^{1}H NMR spectrum for the nanoparticles synthesized with the sodium ω-carboxylate-S-hexyl thiosulfate ligand precursor is shown in Figure S3. The spectrum depicts two broad chemical shifts at 1.54 ppm and 2.19 ppm which correspond to 4 and ~2 hydrogens, respectively. The signals at 1.54 ppm are for the methylene hydrogens in the middle portion of the alkyl chain (δ and γ CH_2 to S). The signal at 2.19 ppm corresponds to the hydrogens α to the carbonyl. The absence of two methylene proton signals (α and β CH_2 signals to the S) indicates the successful covalent binding of the thiolate group to the metal surface. This is also confirmed by the substantial broadening of visible peaks. The absence of sharp peaks confirms that free ligand is not present.

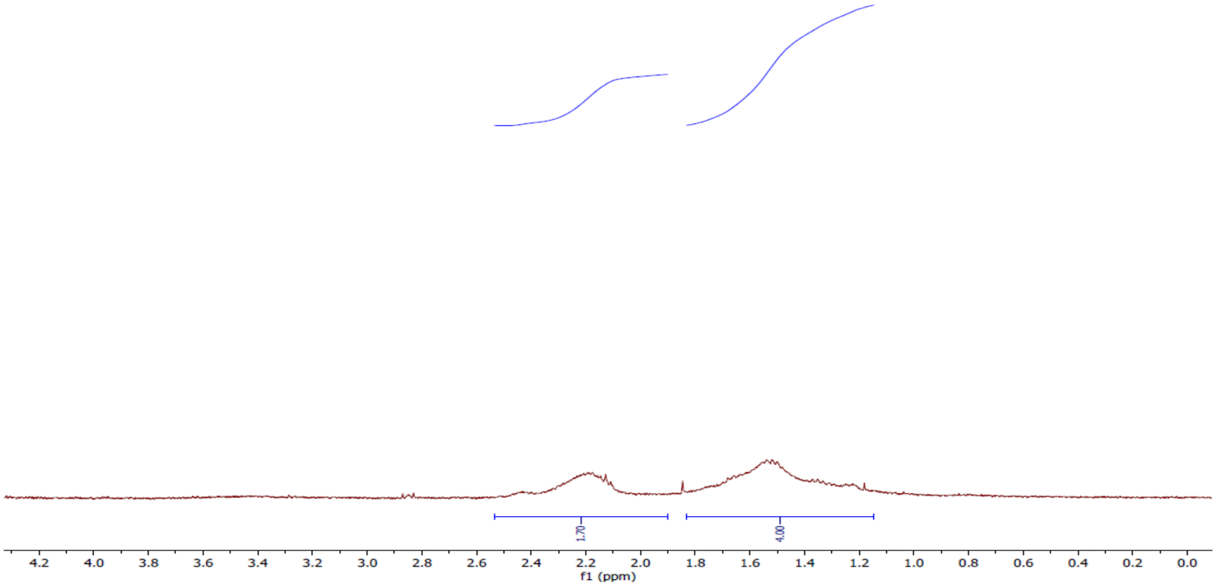

Figure S3. ^{1}H NMR spectrum of 6-(carboxylate)-1-hexanethiolate-capped PdNP.

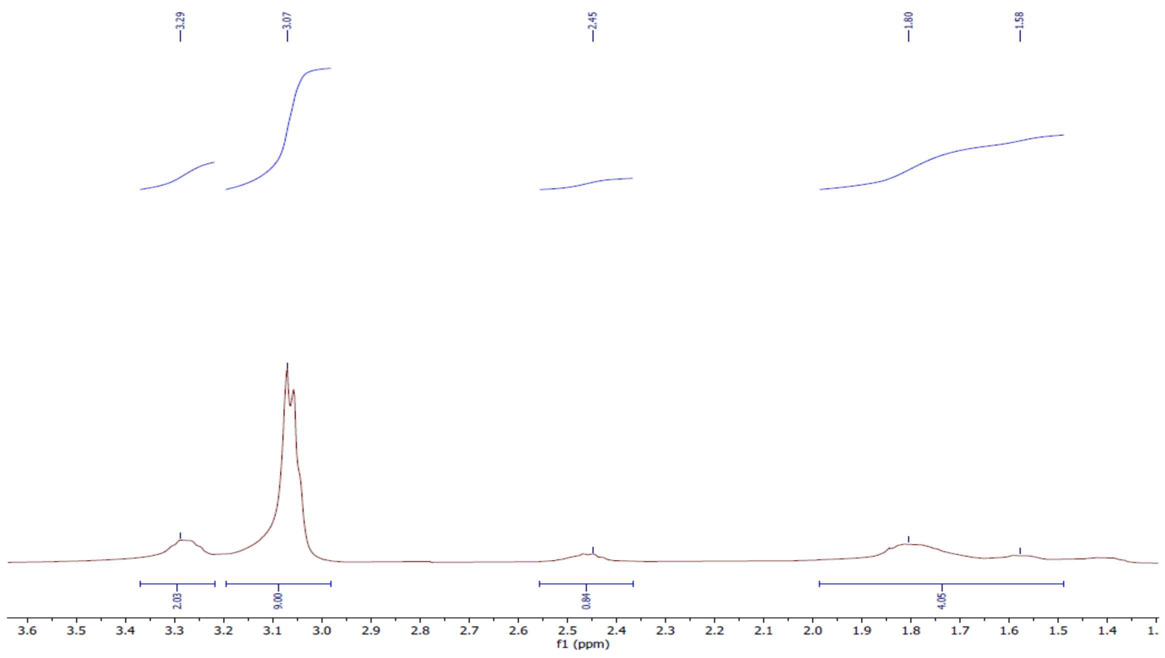

Figure S4. ^{1}H NMR spectrum of 5-(trimethylammonio)pentanethiolate-capped PdNP.

The spectrum in Figure S4 depicts 4 broadened chemical shifts pertaining to the nanoparticle at 1.54 ppm, 1.80 ppm, 3.07 ppm, and 3.29 ppm. The signals at 1.58 ppm and 1.80 ppm are coupled together for a total integration of 4. The signal at 1.58 ppm corresponds to the methylene hydrogens in the middle of the alkyl chain (CH_2 γ to S). The signal at 1.80 ppm pertains to the methylene hydrogens delta to the thiolate group. The signal at 3.07 ppm corresponds to the 9 methyl protons surrounding the nitrogen and the signal at 3.29 ppm corresponds to methylene protons α to the ammonium group. The sharp signal at 3.07 ppm is due to the presence of methyl groups pointing away from the Pd surface, which is allowed to undergo freer movement. Lastly, an additional peak is noted at 2.45 ppm, which is assigned to be solvent contamination (H_2O). The presence of H_2O is also often observed in TGA results (Figures S6 and S7).

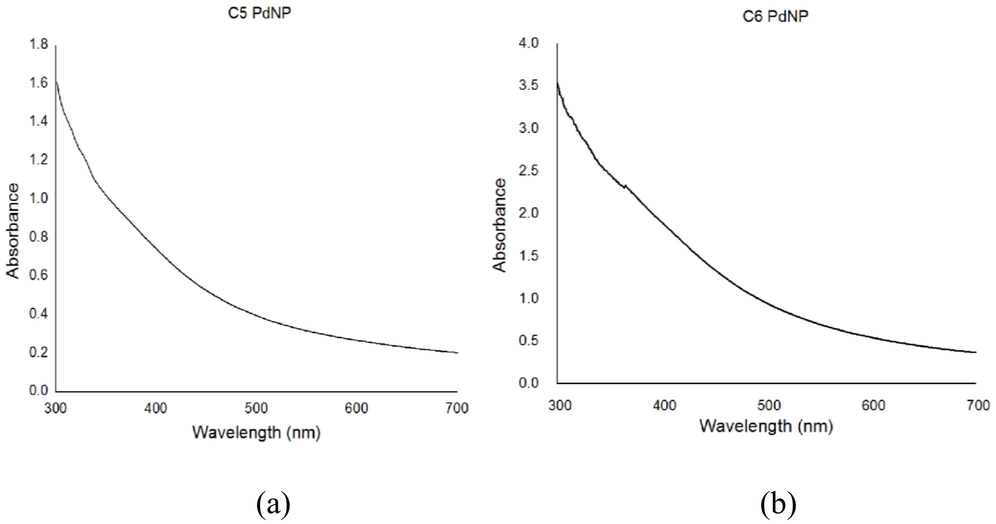

Figure S5. UV-Vis spectra of (a) C5-PdNP (ammonium) and (b) C6-PdNP (carboxylate).

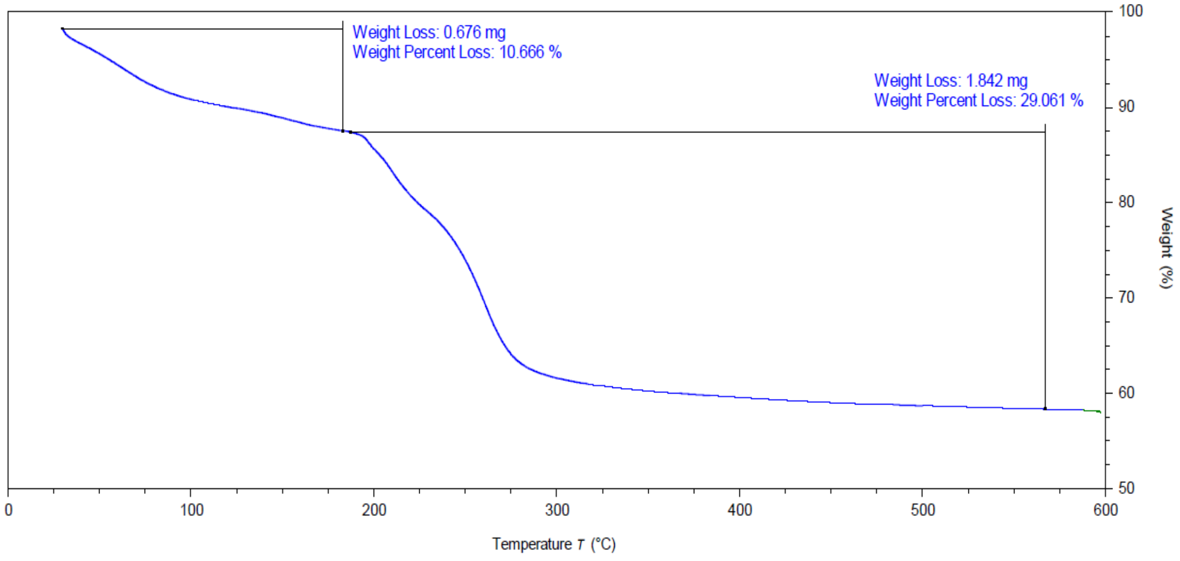

Figure S6. Thermogravimetric analysis of C5-PdNP.

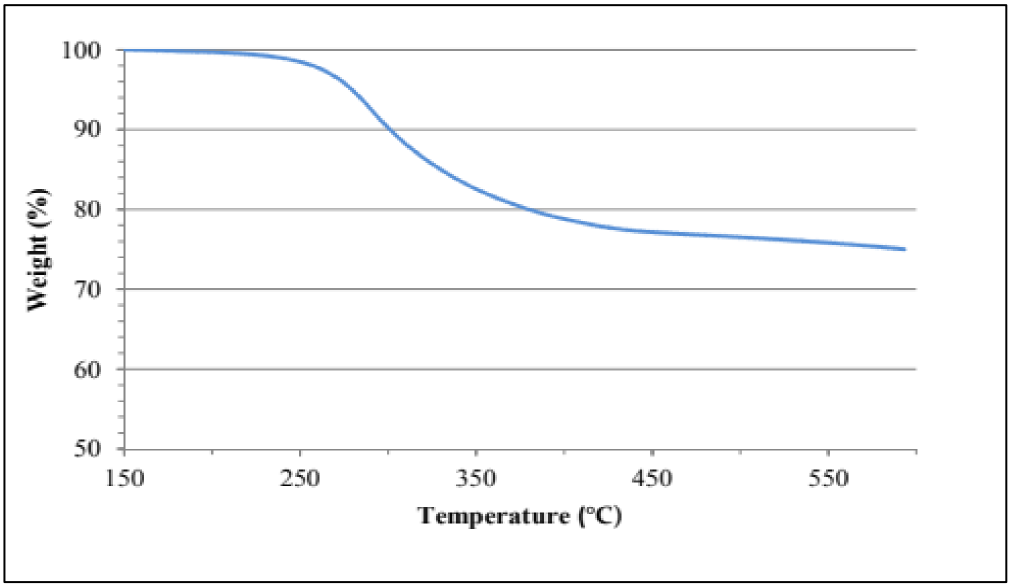

Figure S7. Thermogravimetric analysis of C6-PdNP.

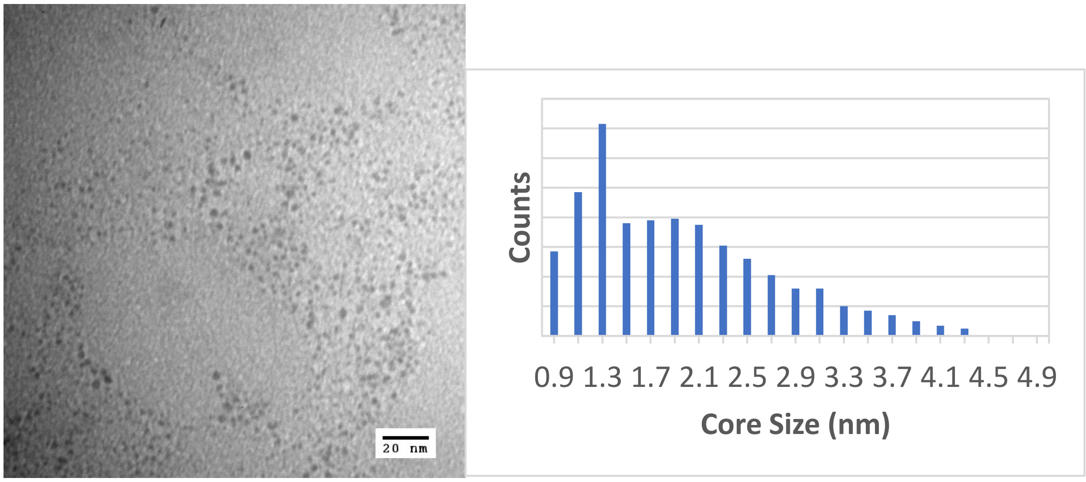

Figure S8. TEM images and size distribution histograms for C6-PdNP.

III. Analysis of PdNP Catalytic Assays

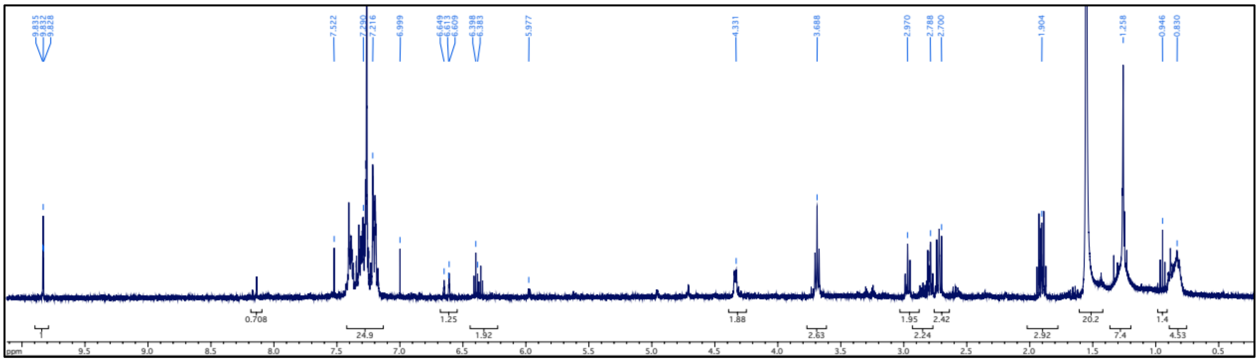

Figure S9: ^{1}H NMR spectrum obtained after reaction of allyl benzene with C6 PdNP in water for 6 h (extracted with $CDCl_3$).

The ^{1}H NMR spectra showed the chemical shifts for both aldehyde and allyl alcohol products indicating the oxidation of terminal alkene carbon. The signal at 9.83 ppm indicates the formation of the aldehyde product which has the hydrogen attached to the carbonyl group. The signal at 2.79 ppm is for the CH_2 hydrogens in the middle portion of the alkyl group (α to the carbonyl group) whereas the signal at 2.97 ppm is for the CH_2 hydrogens at the carbon β to the carbonyl group. For the allyl alcohol, the signal at 4.33 ppm is for the CH_2 hydrogens at the carbon γ to the benzene ring. The signal at 6.39 ppm is for the CH hydrogen in the middle portion of the alkyl group (β to the benzene ring) whereas the signal at 6.61 ppm is for the CH hydrogen at the carbon α to the benzene ring.

The GC spectrum for allyl benzene reaction with C5 PdNP after 24 h is shown in Figure S10. The mass spectra of each peak corresponding to allyl benzene, propyl benzene, benzaldehyde, 1-phenyl-1-propene, and 3-phenylpropanal are shown in Figure S11.

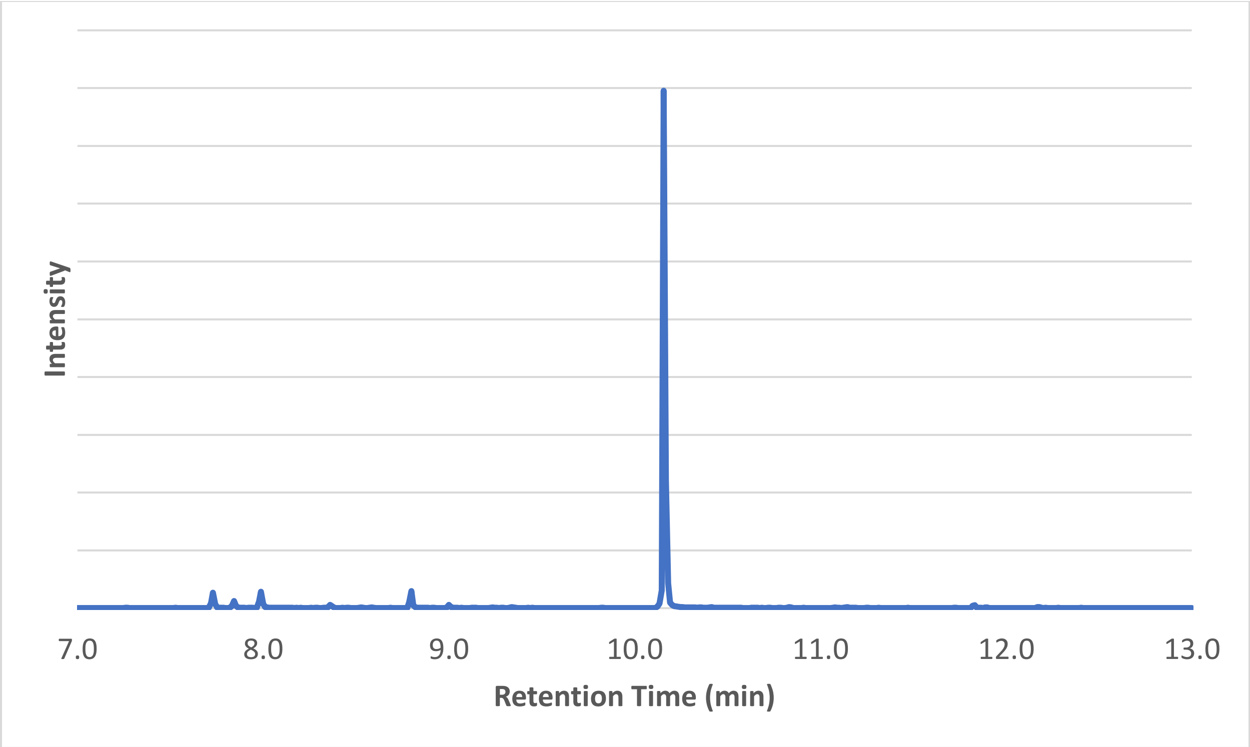

Figure S10: Gas chromatogram obtained after the reaction of allyl benzene with C5 PdNP in water for 24 h (extracted with CH_2Cl_2 and then passed through a pipette filled with silica gel).

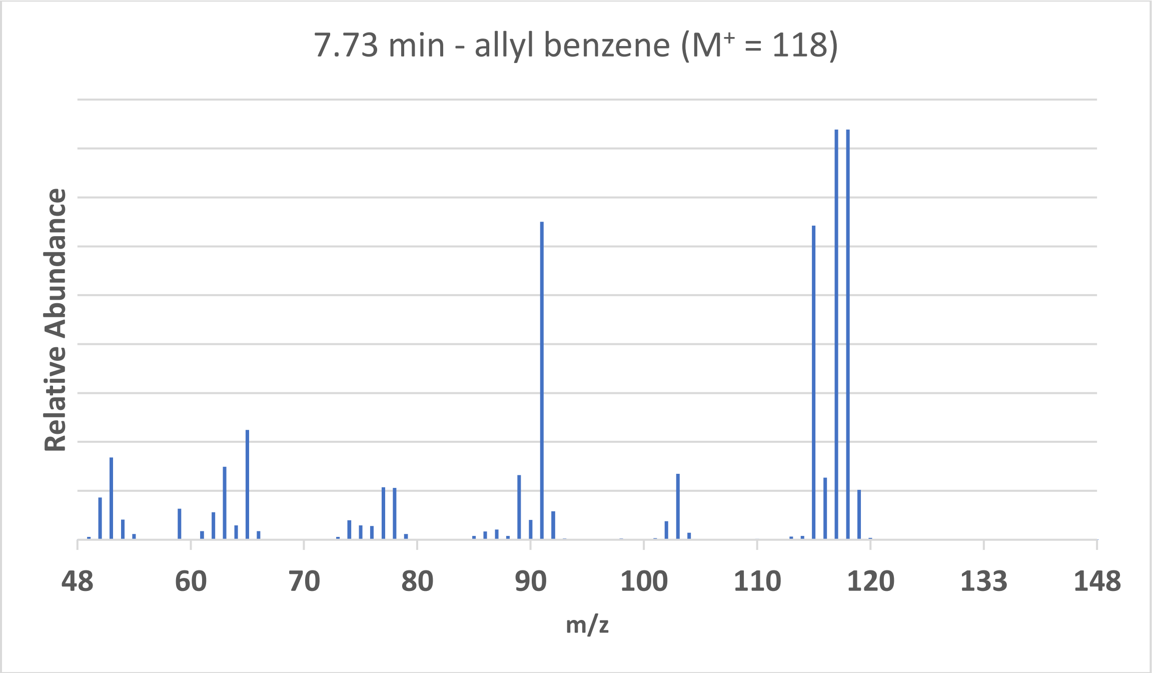

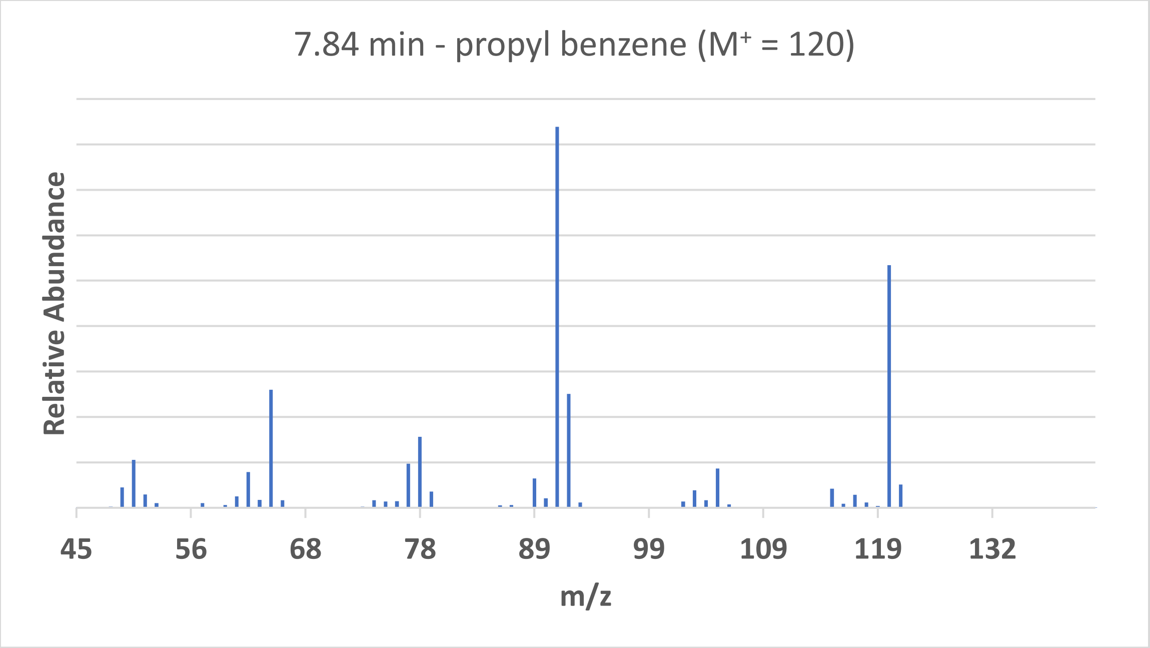
7.84 min - propyl benzene (M+ = 120)
Relative Abundance
45
56
68
78
89
99
109
119
132
m/z

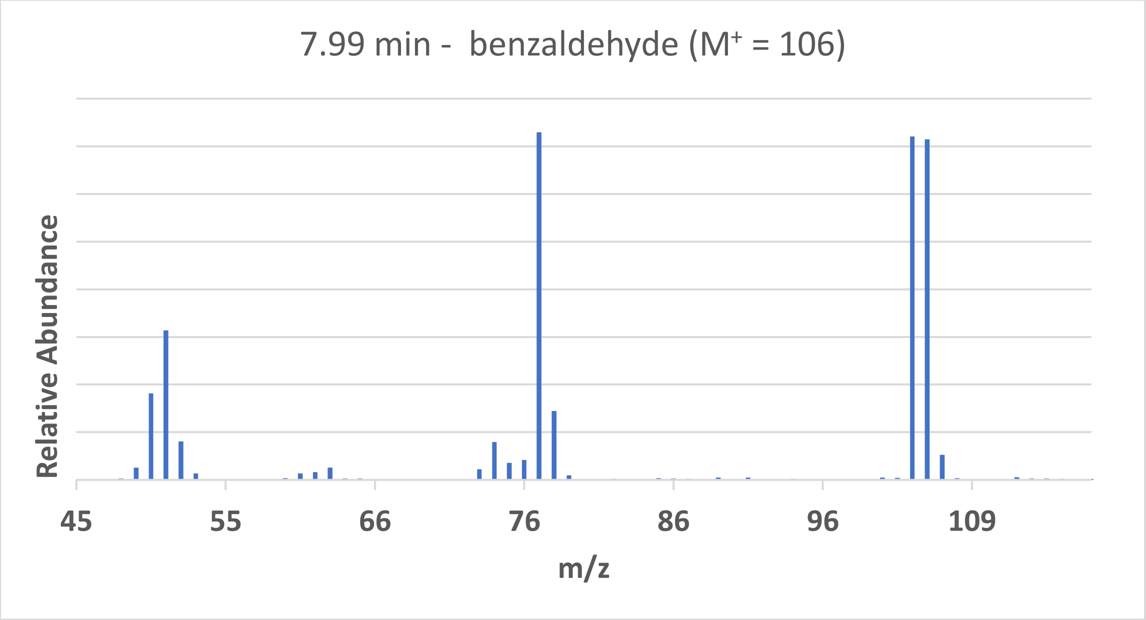
7.99 min - benzaldehyde (M+ = 106)
Relative Abundance
45
55
66
76
86
96
109
m/z

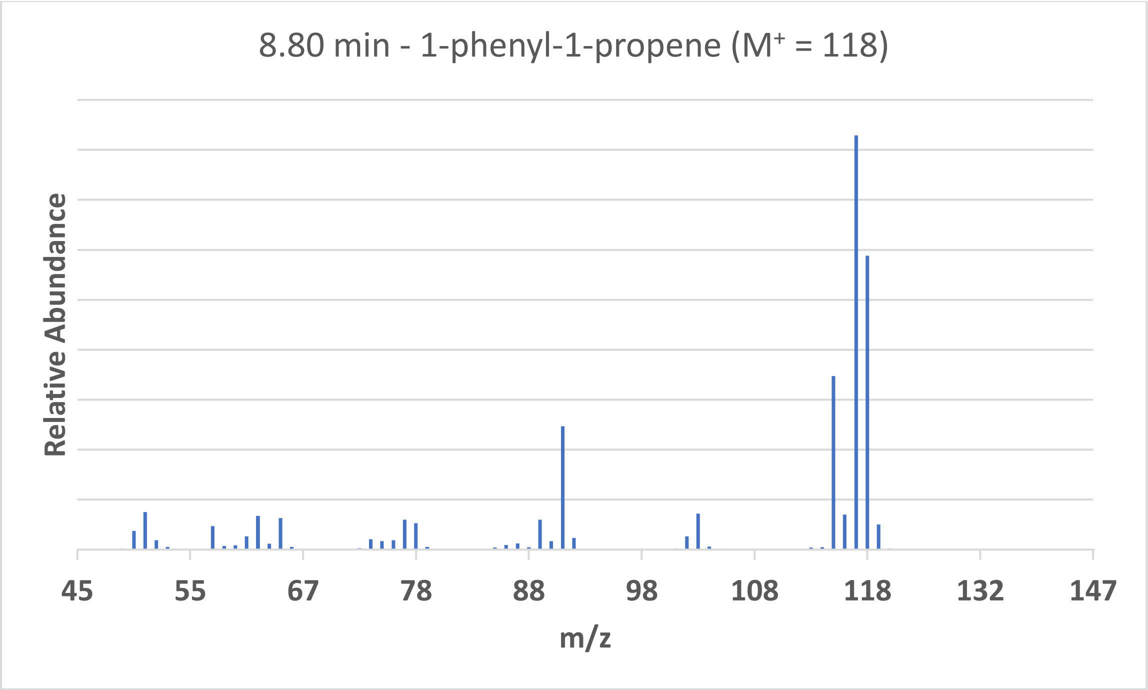

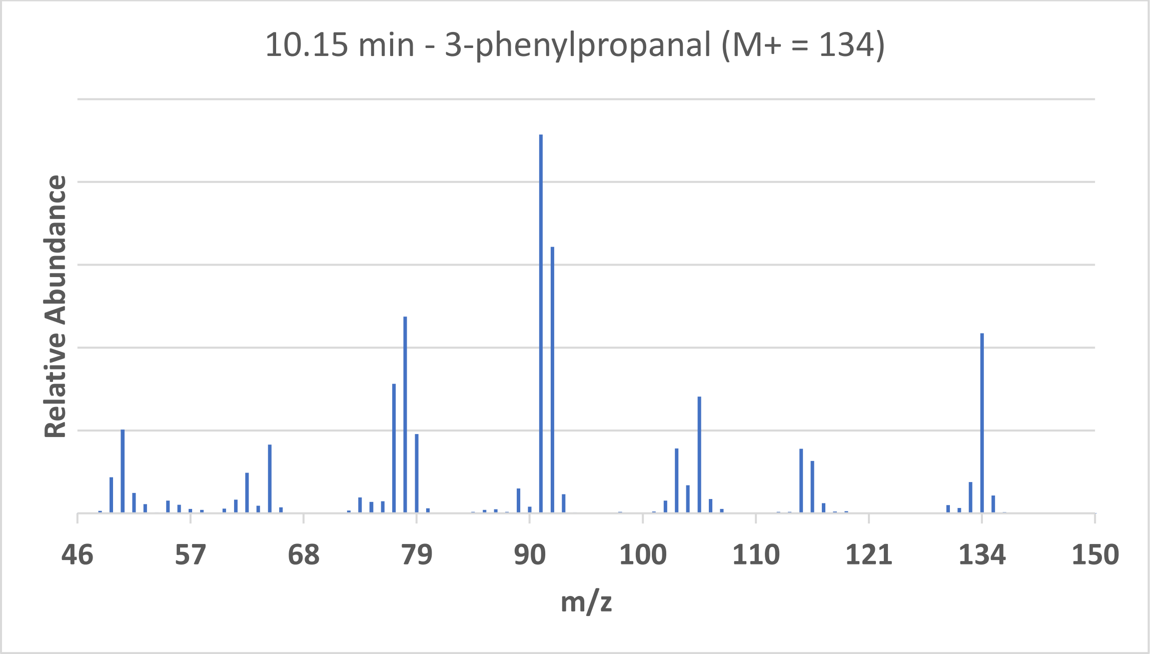

Figure S11: Mass spectra obtained after the reaction of allyl benzene with C5 PdNP in water for 24 h (extracted with CH_2Cl_2 and then passed through a pipette filled with silica gel).

GC-MS spectra showed the additional peaks corresponding to further oxidized products including cinnamaldehyde, 3-phenyl-2-propen-1-ol, and cinnamic acid for some of the reactions of allyl benzene with PdNP (Figure S12).

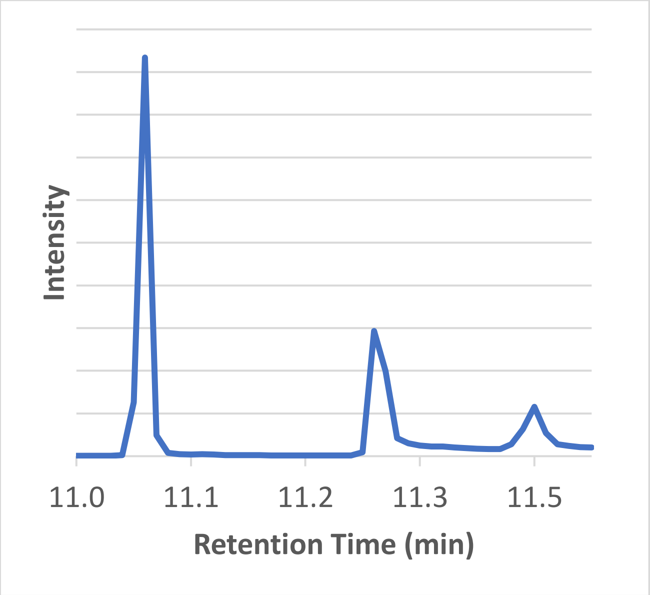

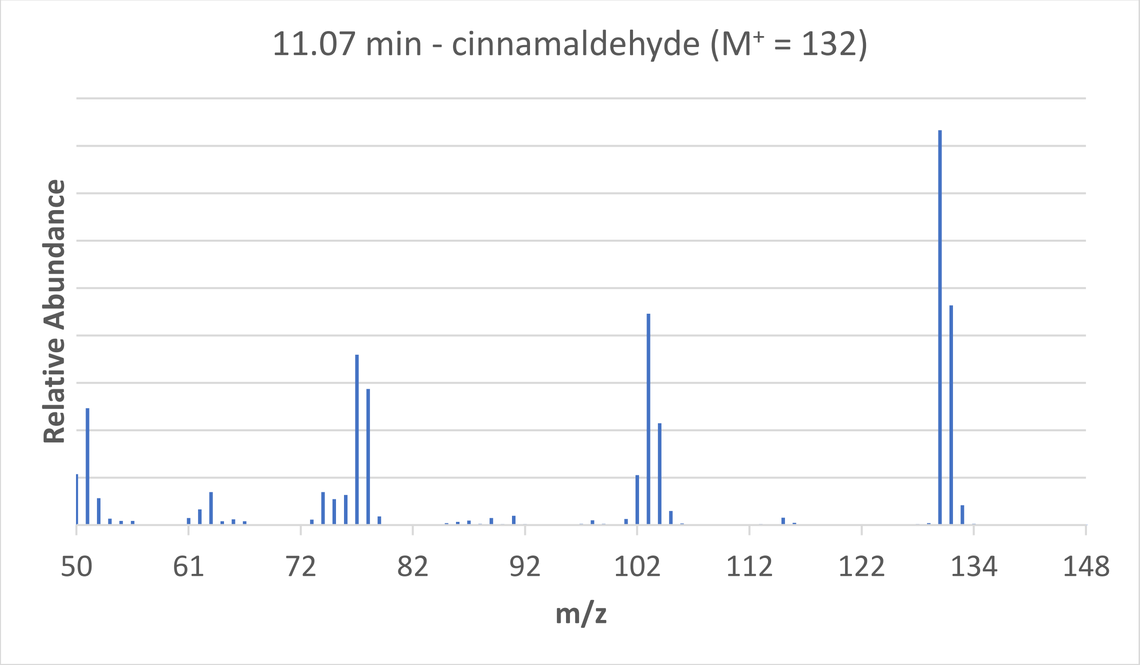

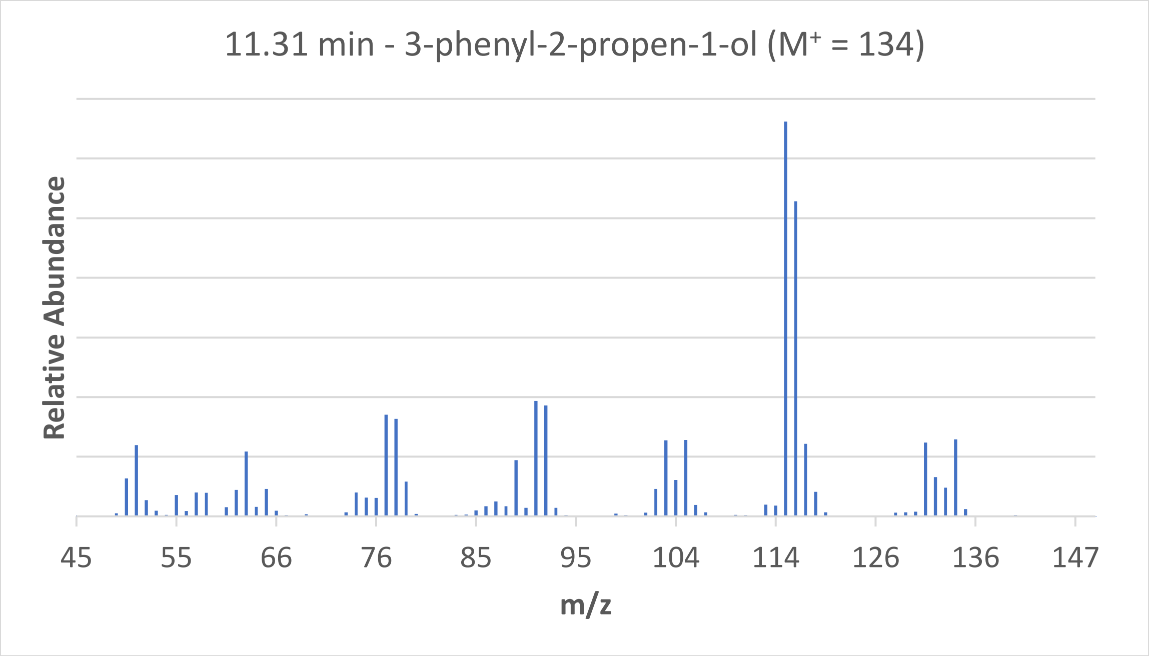

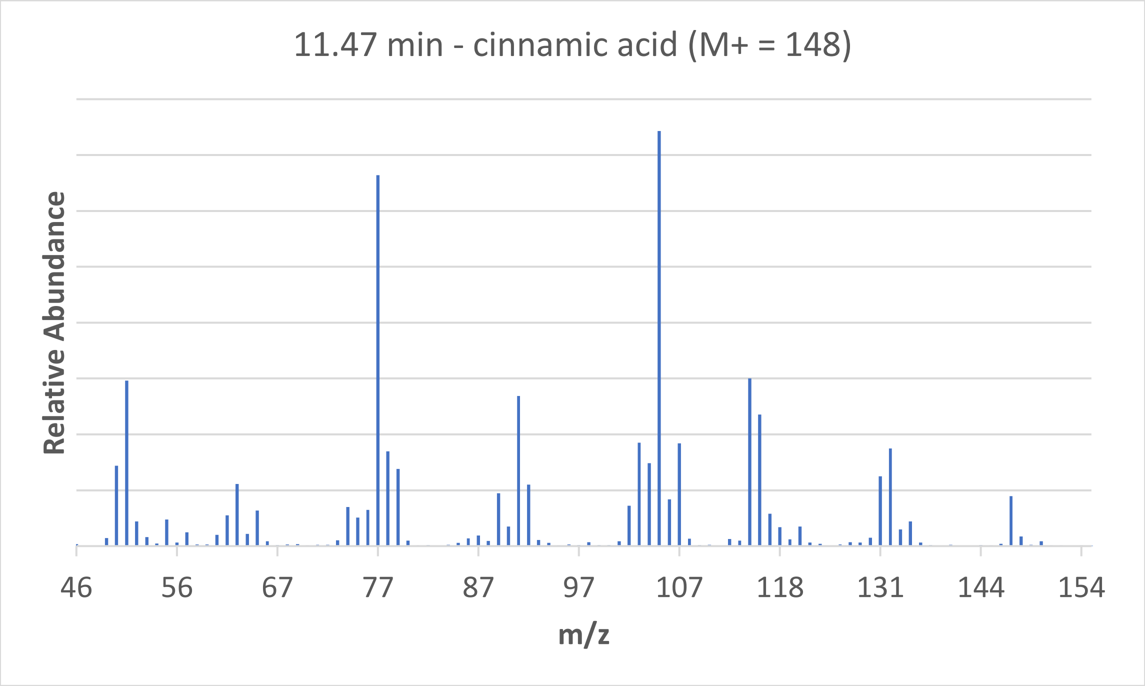

Figure S12: Gas chromatogram and mass spectra obtained after reaction of allyl benzene with C6 PdNP in water for 24 h (extracted with CH_2Cl_2 and then passed through a pipette filled with silica gel).
